# Autoimmune thyroiditis in pregnancy: epidemiology, clinical impact, and contemporary management challenges

**DOI:** 10.25122/jml-2026-0019

**Published:** 2026-06

**Authors:** Alina Potorac, Octavian Munteanu, Alexandru Baros, Elena Evelina Stoica, Monica Mihaela Cirstoiu

**Affiliations:** 1 Doctoral School, Carol Davila University of Medicine and Pharmacy, Bucharest, Romania; 2 Carol Davila University of Medicine and Pharmacy, Bucharest, Romania; 3 Department of Obstetrics and Gynecology, University Emergency Hospital Bucharest, Bucharest, Romania

**Keywords:** maternal thyroid dysfunction, obstetric outcomes, perinatal outcomes, anti-thyroid peroxidase antibodies

## Abstract

Autoimmune thyroiditis represents the most common cause of thyroid dysfunction in women of reproductive age and is frequently encountered during pregnancy. The presence of thyroid autoantibodies, particularly anti-thyroid peroxidase antibodies, has been associated with adverse obstetric outcomes even in the absence of overt thyroid dysfunction. However, optimal screening, monitoring, and treatment strategies remain areas of ongoing debate. This study aimed to review recent evidence on the epidemiology, clinical impact, diagnosis, management, and emerging research directions of autoimmune thyroiditis in pregnancy. A narrative review of observational studies, randomized controlled trials, meta-analyses, and international clinical guidelines published predominantly within the last five years was conducted, focusing on maternal and fetal outcomes, diagnostic approaches, and therapeutic strategies. Autoimmune thyroiditis affects a substantial proportion of pregnant women worldwide and is associated with increased risks of miscarriage, preterm delivery, hypertensive disorders of pregnancy, and postpartum thyroiditis. These associations are observed not only in women with overt or subclinical hypothyroidism but also in euthyroid women with thyroid autoimmunity. While levothyroxine therapy is clearly indicated in overt and selected cases of subclinical hypothyroidism, evidence does not support routine treatment in euthyroid antibody-positive women. Current guidelines recommend targeted screening and close biochemical monitoring rather than universal intervention. Autoimmune thyroiditis in pregnancy is a clinically relevant condition with important implications for maternal and fetal health. A risk-stratified, individualized approach to diagnosis and management is essential, balancing the benefits of treatment against the risks of overtreatment. Further high-quality research is needed to refine risk prediction and identify subgroups that may benefit from targeted therapeutic interventions.

## Introduction

Autoimmune thyroid disease (AITD), which is mostly identified as chronic autoimmune thyroiditis, represents a common organ-specific autoimmune disorder affecting women of childbearing years and represents an important clinical challenge for pregnant patients. As such, AITD is characterized by the presence of thyroid-specific autoantibodies, most commonly anti-thyroid peroxidase (TPO) and anti-thyroglobulin (Tg) antibodies. The clinical manifestations of AITD range from euthyroid autoimmunity through subclinical or overt hypothyroidism [1]. Pregnancy leads to a variety of physiological changes that increase demands for thyroid hormones and alter maternal immune tolerance, which may increase the likelihood of unmasking or worsening of previously undiagnosed autoimmune processes [2,3].

Several studies have examined the incidence of thyroid autoimmunity among pregnant women and report variable rates of thyroid autoimmunity. However, based on studies of different populations and varying serum antibody titers, it appears that the rate of thyroid autoimmunity in pregnant women may be as high as 15–25% [4]. In addition to other factors, there is accumulating evidence that suggests that thyroid autoimmunity, even when thyroid function is normal, is associated with adverse pregnancy outcomes. Specifically, these include increased risk of miscarriage, preterm delivery, gestational hypertension, gestational diabetes mellitus, and fetal growth restriction [2,4,5]. A recent systematic review of existing literature reported that the relative risk of preterm delivery was almost four times greater in pregnant women who were positive for TPO or Tg antibodies compared to those who were negative, and randomized controlled trials indicate that treatment of high-risk pregnant women with levothyroxine resulted in reduced rates of miscarriage and preterm birth [6].

Studies examining potential mechanisms underlying the association between thyroid autoimmunity and adverse pregnancy outcomes propose that immunological disturbances at the maternal-fetal interface and slight reductions in the bioavailability of thyroid hormones may contribute to this association. However, additional research is needed to elucidate causal relationships between the two [7]. Furthermore, prospective cohort studies have demonstrated that thyroid autoimmunity is independently associated with subsequent pregnancy loss, premature rupture of membranes, and hypothyroidism during pregnancy in euthyroid women [8]. Taken collectively, the results of the above-mentioned studies demonstrate the need for early detection and effective management of autoimmune thyroiditis in pregnant women.

## Material and Methods

This study is a narrative review of the most current and relevant scientific evidence, including observational studies, reviews, meta-analyses, and international clinical guidelines. To locate these references, an extensive search of several major electronic databases (PubMed/MEDLINE, Scopus, Web of Science) was conducted, in addition to manually reviewing reference lists. The search strategy utilized keywords or phrases that included “autoimmune thyroiditis,” “thyroid autoimmunity”, “pregnancy”, “anti-thyroid peroxidase antibodies”, and “maternal and fetal outcomes.” To prioritize the highest-quality evidence in conjunction with the most recent data, studies that were recently completed, are of high quality, and include information related to clinical recommendations for diagnosis, clinical impact, and management were prioritized. No formal systematic review protocol was applied, consistent with the narrative design; however, emphasis was placed on selecting studies with robust methodology and clinical relevance.

### Epidemiology of autoimmune thyroiditis in pregnancy

The prevalence of antithyroid antibodies (ATA) varies geographically, ranging from about 5–15% in women of childbearing age, and the same variability occurs in pregnant women. These variations are mainly due to iodine consumption and various other population-specific factors. Some data indicate that iodine availability can vary across regions within a single country, leading to different forms of thyroid autoimmunity. An example of this was found in Denmark. Intra-regionally, there have also been reports of a higher prevalence of thyroid autoantibodies in urban areas than in rural areas, which may be due to environmental and dietary influences. Variability in the reporting of the frequency of ATA may also arise from methodological issues, such as the sensitivity of the assays used to measure them and when they were measured. Therefore, the majority of studies use TPOAb (anti-thyroid peroxidase antibody) and find that individuals testing positive for TPOAb are at much higher rates than those tested for TgAb (anti-thyroglobulin antibody) [9–13].

During pregnancy, there is a shift in the mother’s immune response, characterized by a steady decrease in ATA titer throughout gestation, reaching a nadir in the 3^rd^ trimester, then rebounding after delivery [14]. This temporal pattern may contribute to some of the discrepancies in the literature regarding the prevalence of ATA across trimesters; it also emphasizes the need to define the time point of assessment when evaluating epidemiologic studies.

Several large prospective cohort studies have demonstrated that ATA is an independent predictor of developing thyroid dysfunction during pregnancy, specifically developing subclinical or overt hypothyroidism in previously euthyroid women [15–18]. The increased risk of developing hypothyroidism appears to be related to the length of gestation and appears to be even greater in those women who begin gestation with higher basal TSH levels or coexistent iodine deficiency [19].

At the population level, evidence supports an association between ATA and adverse pregnancy outcomes. Although many of these women have normal serum free thyroid hormone levels, those who test positive for ATA have higher rates of spontaneous abortion, preterm delivery, and postpartum thyroiditis compared with antibody-negative women [2,20,21]. Although the absolute increase in risk for each outcome is low, the high prevalence of ATA makes the overall burden of associated maternal and neonatal morbidity clinically significant.

There are reports of geographical variation in the prevalence of ATA, with higher prevalence in countries where iodine intake is either adequate or excessive, and lower prevalence in countries where iodine intake is inadequate [19,22]. In addition to geography, the presence of other autoimmune diseases, older maternal age, and a first-degree relative with a thyroid disease have all been associated with an increased risk of ATA [2].

In conclusion, ATA represents a significant clinical issue among pregnant women worldwide. It is a major risk factor for the development of gestational thyroid dysfunction and postpartum thyroiditis. Therefore, available evidence provides a strong basis for targeted screening strategies and routine biochemical monitoring in women at increased risk.

### Clinical implications and pregnancy complications associated with autoimmune thyroiditis

Within the past ten years, thyroid autoantibodies have been identified as a separate risk factor for maternal/fetal complications of pregnancy; specifically, they are independent of biochemical thyroid status [15,23]. As such, there are now numerous studies that have demonstrated an association between thyroid autoantibodies and an increased risk of complications associated with pregnancy.

The largest number of studies examining the relationship between thyroid autoantibodies and pregnancy outcomes have demonstrated an association with the risk of pregnancy loss. Studies, including meta-analyses and prospective cohort studies, have shown that ATA positivity is independently associated with an increased risk of pregnancy loss compared with antibody-negative women who remain euthyroid throughout pregnancy [23,24]. The magnitude of this risk varies among studies; however, the association has generally been considered clinically significant.

In addition to an increased risk of pregnancy loss, thyroid autoantibodies have been associated with the development of hypertensive disorders of pregnancy, including gestational hypertension/preeclampsia, as well as gestational diabetes mellitus [15,25,26]. More recently, population-based data have indicated that these associations persist after adjusting for thyroid hormone levels and other traditional risk factors for pregnancy complications [25,26], suggesting that immune-related mechanisms may contribute to placental dysfunction and/or metabolic dysregulation [27].

Women who are ATA-positive are also at increased risk of developing thyroid dysfunction during pregnancy. In particular, the risk of developing subclinical or overt hypothyroidism appears to increase as gestation progresses, particularly in the second and third trimesters [2,10]. Therefore, regular biochemical monitoring of thyroid function during pregnancy is recommended in ATA-positive women.

ATA positivity during pregnancy has also been associated with an increased risk of preterm birth, low birth weight, and small-for-gestational-age (SGA) births. Systematic reviews and meta-analyses have confirmed a statistically significant relationship between thyroid autoantibody positivity and preterm delivery, independent of maternal thyroid hormone concentration [28].

The potential impact of maternal thyroid autoantibody positivity on offspring neurodevelopment has not been established. Earlier observational studies suggested potential associations between maternal thyroid autoantibody positivity and impaired cognitive function. In contrast, more recent evidence indicates that the adverse neurodevelopmental effects of thyroid autoantibody positivity are attributable to maternal hypothyroidism rather than to thyroid autoantibody positivity alone [29,30]. Nevertheless, a potential contribution of immune activation or placental dysfunction cannot be excluded.

Thyroid autoantibody positivity is the primary risk factor for postpartum thyroiditis (PPT) and may occur in approximately 30–50% of thyroid autoantibody-positive women. PPT can manifest in several forms, including transient thyrotoxicosis, hypothyroidism, or a biphasic course, and a significant portion of women will develop permanent hypothyroidism over time (Table 1) [31].

**Table 1:** Maternal and fetal risks associated with thyroid autoantibody positivity in pregnancy

Risk category	Key findings	Strength/nature of evidence	Clinical implications
**Independent risk factor**	Thyroid autoantibodies constitute an independent risk factor for maternal and fetal complications, irrespective of biochemical thyroid status	Observational studies over the past decade, population-based analyses	Risk assessment should not rely solely on thyroid hormone levels [8,23]
**Pregnancy loss**	Increased risk of miscarriage in thyroid autoantibody–positive, euthyroid women compared with antibody-negative women	Meta-analyses and prospective cohort studies	Clinically significant increased risk; warrants closer surveillance [23,24]
**Hypertensive disorders of pregnancy**	Association with gestational hypertension and preeclampsia	Population-based studies adjusting for thyroid function and traditional risk factors	Suggests immune-mediated placental dysfunction [25,26]
**Gestational diabetes mellitus**	Increased risk of gestational diabetes	Population-based studies with multivariable adjustment	Indicates possible immune-related metabolic dysregulation [25]
**Thyroid dysfunction during pregnancy**	Higher risk of developing subclinical or overt hypothyroidism, particularly in the second and third trimesters	Longitudinal observational studies	Regular biochemical monitoring of thyroid function is recommended [2,10,15]
**Preterm birth and fetal growth**	Increased risk of preterm birth, low birth weight, and small-for-gestational-age infants	Systematic reviews and meta-analyses, independent of thyroid hormone levels	Highlights the need for obstetric risk stratification [28]
**Offspring neurodevelopment**	No definitive independent effect established; earlier studies suggested cognitive impairment, whereas recent evidence attributes risk primarily to maternal hypothyroidism	Observational studies with evolving interpretations	Immune or placental effects cannot be fully excluded [29,30]
**Postpartum thyroiditis**	Primary risk factor for postpartum thyroiditis, occurring in approximately 30–50% of antibody-positive women; risk of permanent hypothyroidism	Long-term cohort studies	Postpartum thyroid function follow-up is essential [31]

### Diagnosis, treatment, and management

The clinical and biochemical assessment of pregnant women with autoimmune thyroiditis (AIT) requires a comprehensive understanding of the physiological adaptations occurring during pregnancy and the potential effects of thyroid autoimmunity on both maternal and fetal health. Properly interpreting thyroid function testing and making appropriate therapeutic decisions are crucial in preventing either over-treating or under-treating pregnant women.

Serum levels of thyroid-stimulating hormone (TSH) and free thyroxine (FT4) are used to assess thyroid function in pregnant women, using trimester-specific reference values when available [32]. Due to the physiological adaptations that occur during pregnancy, the normal physiology of the thyroid gland can be altered; these include a decrease in TSH due to the suppressive effect of human chorionic gonadotropin (hCG) in the early stages of pregnancy and an increase in thyroxine production later in pregnancy, which can make early detection of thyroid dysfunction difficult [33].

The diagnosis of autoimmune thyroiditis is confirmed by the presence of thyroid autoantibodies (TPOAb and/or TgAb). TPOAb is the most clinically useful antibody for predicting the development of hypothyroidism during pregnancy and an increased risk of postpartum thyroiditis [34]. The use of ultrasound to evaluate the thyroid gland is not routinely indicated during pregnancy, except in rare cases of diagnostic ambiguity [32].

Screening for thyroid dysfunction and autoimmunity is not universally recommended for all pregnant women. Still, it is typically limited to women at risk for having thyroid problems, such as having a personal or family history of thyroid disease, being afflicted with other autoimmune diseases, experiencing difficulties with conception, having recurrent spontaneous abortions, or experiencing clinical manifestations indicative of thyroid dysfunction [32].

Decisions regarding the management and treatment of AIT during pregnancy are typically based on the pregnant woman’s thyroid function rather than on the presence of thyroid autoantibodies.

Women with overt hypothyroidism (defined as abnormal elevation in TSH and reduction in FT4) will require LT4 replacement therapy. Untreated hypothyroidism has been shown to significantly impact the health of the mother and fetus, including increased risks of miscarriage, preeclampsia, premature delivery, and impaired neurodevelopment of the fetus [32,35]. Women diagnosed with overt hypothyroidism should begin receiving LT4 therapy immediately, with regular assessments to maintain TSH levels consistent with the trimester of pregnancy. Thyroid function testing should be performed approximately every 4–6 weeks during pregnancy [33].

Women diagnosed with subclinical hypothyroidism (elevated TSH with normal FT4) and positive for TPOAb typically warrant initiation of LT4 replacement therapy if their TSH levels exceed the upper reference limit for the trimester of pregnancy. Studies suggest that treatment of women with subclinical hypothyroidism may potentially reduce the risk of pregnancy loss and progression to overt hypothyroidism; however, the amount of benefit obtained through therapy in this group has varied widely among various studies [32,36].

The treatment of euthyroid women pregnant with thyroid autoimmunity continues to be an area of considerable debate. Several recent randomized controlled trials and meta-analyses have failed to demonstrate a clear benefit of administering routine LT4 supplementation to euthyroid pregnant women with thyroid autoimmunity to reduce the rates of miscarriage or preterm delivery [31,36]. Most current guidelines recommend against routine treatment of euthyroid pregnant women with thyroid autoimmunity but advocate for careful biochemical monitoring, particularly during the first half of pregnancy, when the risk of thyroid function deterioration is greatest [37].

Pregnant women with thyroid autoimmunity are also at an increased risk of developing postpartum thyroiditis, which may manifest as transient hyperthyroidism, hypothyroidism, or a biphasic course. Therefore, postpartum evaluation of thyroid function is suggested, particularly in women who were TPOAb positive during pregnancy or who received LT4 therapy [2,34]. Furthermore, long-term follow-up is suggested, as many women with thyroid autoimmunity will develop permanent hypothyroidism [32].

In general, the treatment of autoimmune thyroiditis during pregnancy should focus on identifying individual risk, avoiding unnecessary treatment for low-risk euthyroid women, and providing timely treatment for women identified as having thyroid dysfunction. Although the treatment of overt and subclinical hypothyroidism during pregnancy is well supported, the optimal management of isolated thyroid autoimmunity continues to evolve, supporting the need for additional large-scale, well-designed clinical trials.

### Recommendations from current clinical practice guidelines

Clinical practice guidelines concerning AIT in pregnancy generally concur in their overall recommendations; however, each set of guidelines acknowledges the extent of uncertainty regarding the management of euthyroid women with thyroid autoimmunity. The most influential clinical practice guidelines are those of the American Thyroid Association, the European Thyroid Association, and other international expert consensus statements [23,38,39].

No clinical practice guideline recommends universal screening for thyroid autoimmunity in pregnancy. Each clinical practice guideline supports a targeted screening strategy for women at increased risk, such as a personal or family history of thyroid disease, autoimmune diseases, infertility, recurrent pregnancy loss, preterm delivery, or clinical symptoms indicative of thyroid dysfunction. No clinical practice guidelines recommend routine testing for thyroid autoantibodies in low-risk, asymptomatic pregnant women, due to a lack of definitive evidence that early detection and treatment improve outcomes in this population [38,39].

There is significant consensus among clinical practice guidelines that all cases of overt hypothyroidism in pregnancy should be treated immediately with LT4, regardless of the underlying cause of the hypothyroidism. Treatment of hypothyroidism in pregnancy aims to restore maternal thyroid hormone levels to normal and to reduce the risk of adverse maternal and fetal outcomes, including miscarriage, hypertension, preterm birth, and impaired neurocognitive development [40].

The recommendations for the treatment of subclinical hypothyroidism in pregnancy are less uniform. Both the American Thyroid Association and European Thyroid Association clinical practice guidelines support the use of LT4 in pregnant women who are TPOAb-positive and have elevated TSH concentrations greater than the pregnancy-specific reference values, especially if TSH > 4.0 mIU/L. The rationale behind these recommendations is based upon observational studies, which have demonstrated a relationship between untreated subclinical hypothyroidism and adverse pregnancy outcomes and also an increased likelihood of progression to overt hypothyroidism [38,39].

The management of euthyroid pregnant women with thyroid autoantibodies is the most controversial issue in the clinical practice guidelines. Numerous studies, including randomized controlled trials and meta-analyses, have been conducted over the last decade. These studies have not consistently demonstrated a reduction in miscarriage or preterm delivery rates in euthyroid pregnant women receiving LT4 supplements [38,41]. Therefore, routine LT4 supplementation in euthyroid pregnant women with positive thyroid autoantibodies is not recommended by clinical practice guidelines. Instead, the emphasis in clinical practice guidelines is on close monitoring of biochemistry, especially during the first two trimesters of pregnancy, when thyroid hormone requirements are at their greatest, and the risk of hypothyroidism is highest. Recommendations include periodic assessment of TSH (every 4-6 weeks) in antibody-positive women, even when baseline thyroid function tests indicate euthyroid status [38,39].

All major clinical practice guidelines emphasize the need for postpartum surveillance in women with thyroid autoimmunity, because of the high risk of postpartum thyroiditis and subsequent permanent hypothyroidism. Recommendations include thyroid function testing in the first 6 to 12 months after delivery, especially in women who had TPOAb positivity during pregnancy or were receiving LT4 therapy [39,42].

In general, current clinical practice guidelines recommend a risk-based, individually tailored approach to managing autoimmune thyroiditis in pregnancy. There is considerable evidence supporting the treatment of overt and selected cases of subclinical hypothyroidism; however, the management of isolated thyroid autoimmunity is conservative and reflects the current level of uncertainty and the need for additional high-quality clinical trials.

### Future perspectives

Future directions and perspectives regarding autoimmune thyroiditis during pregnancy remain numerous and will guide future investigations to address the remaining gaps in knowledge. The primary areas of concern include molecular mechanisms, predictive biomarkers, therapeutic innovation, and long-term outcomes.

The molecular and immunological mechanisms that define the onset and progression of AIT in pregnant women have not been fully delineated. The interplay among genetic predisposition, epigenetic modification, environmental exposure, and immune dysregulation, which defines both disease initiation and progression, has recently begun to emerge [2,6]. Epigenetic modifications, such as DNA methylation and noncoding RNAs, appear to play a critical role in converting environmental exposures into altered immune responses in genetically predisposed individuals [43].

Autoimmune thyroid antibodies (i.e., TPOAb) are commonly used to assess the presence of autoimmune thyroiditis in pregnancy; however, they are insufficiently specific for predicting adverse outcomes and/or disease progression. Therefore, future research should focus on developing multi-biomarker panels that integrate autoantibody profiles with immune signatures, cytokine profiles, and metabolic markers [44], using high-throughput “omics” methodologies to develop individualized risk stratification models that go beyond traditional serologic methods.

Levothyroxine remains the primary treatment for overt and clinically relevant subclinical hypothyroidism during pregnancy; however, there are ongoing explorations of other therapeutic options. Some preliminary evidence exists that certain micronutrient and immunomodulatory treatments may influence thyroid autoimmunity. For example, selenium appears to modulate the immune response and decrease levels of thyroid autoantibodies; however, there is currently limited evidence, and additional rigorous trial-based evidence is required to definitively establish whether selenium exerts beneficial effects on maternal/fetal outcomes [45,46]. There is considerable potential to understand the effects of nutritional and immunoregulatory interventions on maternal and fetal outcomes, leading to the development of new adjunctive treatment modalities.

Recent data suggest that the gut microbiota plays a role in the etiology of autoimmune thyroid diseases and their systemic effects. Disruption of the gut microbiota (dysbiosis) has been linked to alterations in immune regulation and autoimmune activity, indicating that modulation of the microbiome may represent a novel therapeutic option [47]. Further research investigating the pregnancy-specific gut-thyroid axis and its relationship to systemic immunity and pregnancy outcomes remains in its infancy and offers a promising area for future study.

Thyroid autoimmunity may also influence fertility outcomes and success rates in assisted reproductive technologies (ART). Recent narrative reviews suggest potential effects of thyroid autoimmunity on ovarian reserve and embryo implantation; however, evidence is still inconclusive [47,48]. Prospective studies planned to investigate the relationship between thyroid autoimmunity and ART-related fertility outcomes should provide clarity regarding mechanisms and optimal pre-ART screening and management protocols for women with thyroid autoimmunity.

Although immediate obstetric outcomes of thyroid autoimmunity are well-established, the long-term health consequences for both mothers and their children exposed to maternal thyroid autoimmunity are poorly understood.

Advancements in this field will benefit from large collaborative research networks and integrated data platforms combining clinical, biochemical, and molecular data across diverse populations. Data integration through individual-participant data meta-analysis and the creation of harmonized registry databases can increase statistical power and enable robust subgroup analyses to identify high-risk phenotypes and refine intervention thresholds (Table 2).

**Table 2:** Future research domains in autoimmune thyroiditis during pregnancy

Focus area	Key issues and knowledge gaps	Emerging approaches	Potential clinical impact
**Molecular and immunological mechanisms**	Incomplete understanding of disease initiation and progression during pregnancy; complex interactions among genetic susceptibility, epigenetic regulation, environmental exposures, and immune dysregulation	Investigation of DNA methylation, noncoding RNAs, and immune signaling pathways using molecular and systems biology approaches	Identification of novel therapeutic targets and improved prediction of disease trajectory
**Predictive biomarkers**	Thyroid autoantibodies (e.g., TPOAb) lack specificity for predicting adverse pregnancy outcomes or disease progression	Development of multi-biomarker panels integrating autoantibodies, immune signatures, cytokines, and metabolic markers using high-throughput “omics” technologies	Individualized risk stratification beyond conventional serologic testing
**Therapeutic innovation**	Limited treatment options beyond levothyroxine for hypothyroidism; unclear benefit of adjunctive therapies	Evaluation of micronutrient supplementation (e.g., selenium) and immunomodulatory interventions in rigorously designed clinical trials	Potential development of adjunctive therapies to modify autoimmunity and improve maternal/fetal outcomes
**Nutritional and immunoregulatory interventions**	Insufficient evidence regarding the impact of nutritional modulation on thyroid autoimmunity and pregnancy outcomes	Targeted trials assessing nutritional and immune-modifying strategies	Expansion of non-pharmacologic treatment strategies
**Gut–thyroid axis**	Role of gut microbiota in AIT during pregnancy remains poorly defined	Research into pregnancy-specific gut microbiome alterations and immune regulation	Novel microbiome-based therapeutic or preventive strategies
**Fertility and assisted reproductive technologies (ART)**	Uncertain impact of thyroid autoimmunity on ovarian reserve, implantation, and ART success rates	Prospective studies examining fertility outcomes and pre-ART screening strategies	Optimization of fertility counseling and management in women with thyroid autoimmunity
**Long-term maternal and offspring outcomes**	Limited data on long-term health effects of maternal thyroid autoimmunity on mothers and children	Longitudinal cohort studies with extended follow-up	Improved understanding of lifelong health risks and preventive strategies
**Collaborative research and data integration**	Fragmented data and limited power for subgroup analyses	Large-scale collaborative networks, harmonized registries, and individual participant data meta-analyses	Identification of high-risk phenotypes and refinement of intervention thresholds

## Conclusion

Autoimmune thyroiditis is a very common issue in pregnancy, and it is associated with an increased risk of developing gestational thyroid dysfunction, poor obstetrical outcomes, and postpartum thyroid disease. The growing body of evidence shows that having a history of thyroid autoantibodies can result in clinical risks even when a woman’s thyroid function levels are normal; this is contrary to the previous belief that being biochemically euthyroid was sufficient to prevent the development of adverse outcomes.

The current body of literature supports the immediate treatment of overt hypothyroidism and certain cases of subclinical hypothyroidism during pregnancy; however, there is currently no justification for routinely treating euthyroid pregnant women with thyroid autoimmunity with levothyroxine. Therefore, contemporary practices focus on the selective screening of pregnant women at high risk of thyroid dysfunction and their subsequent individualized risk assessments and biochemical monitoring as opposed to universal therapy.

It is anticipated that future research will provide additional information regarding the identification of biomarkers for improved risk stratification in pregnant women who have a history of thyroid autoimmunity. Additionally, it is anticipated that future research will provide greater insight into the mechanisms by which thyroid autoimmunity affects both the fetus and the mother, and into how to develop more effective personalized therapies. In the absence of these data, a conservative, evidence-based, multi-disciplinary approach will continue to be the best way to optimize both the maternal and fetal outcomes in pregnancies complicated by autoimmune thyroiditis.
